# Research progress of enamel matrix derivative on periodontal tissue regeneration: a narrative review

**DOI:** 10.3389/fdmed.2025.1611402

**Published:** 2025-06-30

**Authors:** Chenyang Xiang, Linglin Zhang, Enfu Tao

**Affiliations:** ^1^ Department of Stomatology, Wenling Maternal and Child Health Care Hospital, Wenling, Zhejiang, China; ^2^ State Key Laboratory of Oral Diseases & National Clinical Research Center for Oral Diseases, Sichuan University, Chengdu, Sichuan, China; ^3^ Department of Conservative Dentistry and Endodontics, West China Hospital of Stomatology, Sichuan University, Chengdu, Sichuan, China; ^4^ Department of Neonatology and NICU, Wenling Maternal and Child Health Care Hospital, Wenling, Zhejiang, China

**Keywords:** enamel matrix derivative, periodontal regeneration, periodontitis, dental implantation, tooth replantation, Wnt/β-catenin signaling pathway

## Abstract

Extensive research has demonstrated that enamel matrix derivative (EMD) facilitates periodontal tissue regeneration, enabling the genuine regeneration of cementum, periodontal ligament, and alveolar bone. Its clinical formulation, Emdogain, is currently employed in the treatment of alveolar bone defects resulting from periodontitis, as well as in dental implantation and tooth replantation procedures. This review aims to synthesize recent findings on the application of EMD in periodontology, with a particular emphasis on its efficacy in addressing alveolar bone defects, peri-implantitis, and related conditions. Furthermore, this review examines the influence of EMD on the proliferation and differentiation of periodontal ligament stem cells, bone marrow stem cells, osteoblasts, and fibroblasts. It also assesses the secretion of various growth factors, including transforming growth factor-β1 (TGF-β1), bone morphogenetic protein-2 (BMP-2), collagen type 1 (COL-1), runt-related transcription factor 2 (RUNX2), and osteocalcin (OCN). Additionally, the review seeks to identify the optimal concentration for EMD application. Collectively, the studies reviewed herein suggest that EMD significantly enhances the proliferation and differentiation of relevant cellular components. The optimal concentration of EMD varies by environment and cell type. In minimally invasive periodontal surgery for intrabony defects, EMD enhances periodontal health, gingival recession coverage, and bone filling. It also benefits open-flap debridement and non-surgical treatments. However, EMD offers no extra benefits for Class II furcation defects. In treating gingival recession with coronally advanced flap (CAF) and subepithelial connective tissue graft (SCTG), EMD significantly boosts root coverage, but not with the modified coronally advanced tunnel (MCAT) technique or the semilunar coronally advanced flap. EMD's anti-inflammatory and immunomodulatory properties reduce inflammation around implants. This review indicates that EMD shows potential for periodontal regeneration, but more randomized clinical trials are necessary to assess its effectiveness.

## Introduction

Substantial evidence indicates that EMD can effectively promote the regeneration of periodontal tissues, including cementum, periodontal ligament, and alveolar bone, particularly in cases involving alveolar bone defects (1–5). Histological analyses have demonstrated the presence of functionally oriented periodontal ligament fibers within newly formed cementum and alveolar bone, exhibiting morphological and biological characteristics akin to natural periodontal tissues (6, 7). These findings strongly endorse the clinical application of EMD, presenting innovative therapeutic strategies for the treatment of periodontitis. Nonetheless, standardized protocols concerning the optimal delivery methods and concentrations of EMD have yet to be established. Ongoing research continues to investigate the applications of EMD for alveolar bone defects, with emerging studies exploring their potential use in dental implantation and tooth replantation. In light of the necessity to integrate recent advancements into clinical practice, an updated review of this field is imperative.

EMD are specialized proteins secreted by Hertwig's epithelial root sheath during the process of tooth development. These proteins exhibit a complex composition, predominantly consisting of amelogenin (constituting over 90% of the total protein content), along with enamelin, ameloblastin, proteases, and various growth factors (8). The significant evolutionary conservation of Am genes across various species, such as the notable homology between porcine and human Am, has prompted researchers to frequently purify EMD from young pig tooth germs through acetic acid extraction. Empirical studies have demonstrated that EMD facilitates periodontal tissue regeneration by promoting new attachment formation during tooth development (9). A retrospective cohort study spanning ten years has shown that the clinical improvements achieved through EMD-mediated regeneration can be sustained over the long term (10). Further research has elucidated EMD's anti-inflammatory properties (11, 12), its capacity to enhance local growth factor expression and angiogenesis (13), and its potential to direct dental pulp stem cell differentiation towards odontoblastic lineages (14). The Swedish-developed commercial product Emdogain®, which combines EMD with a carrier gel, has gained widespread use in both dental research and clinical practice. Nevertheless, certain studies have reported suboptimal clinical outcomes associated with the application of EMD. This review aims to critically evaluate contemporary clinical research findings concerning the efficacy of EMD in periodontal therapy and its other applications. Additionally, it seeks to identify potential factors contributing to these unsatisfactory results, thereby offering enhanced guidance for clinical practice.

## Effects of enamel matrix derivative on periodontal regeneration-related cells

**Figure 1:**
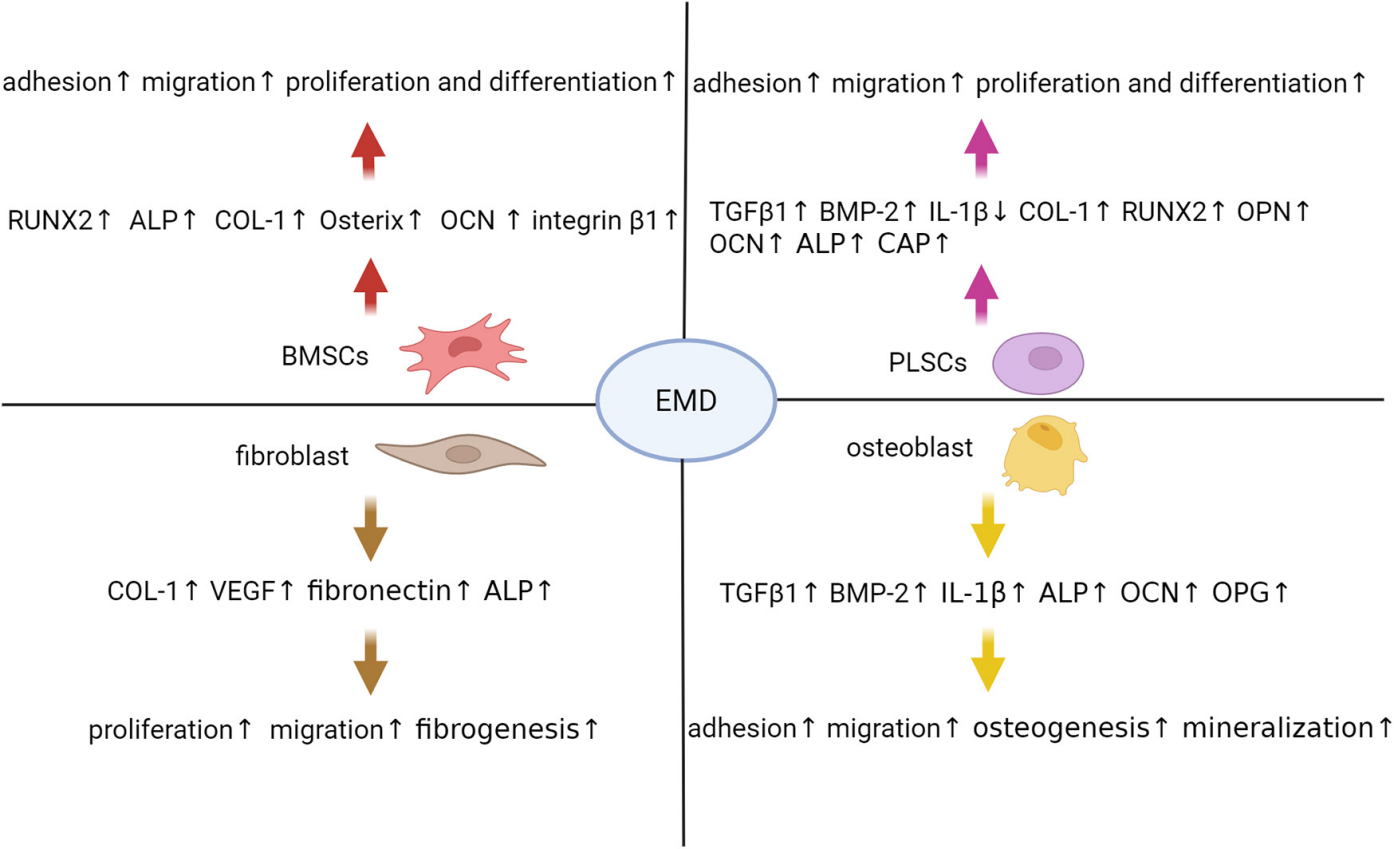
Enamel matrix derivative modulates multiple cellular processes critical for periodontal regeneration. Extensive research has demonstrated that EMD exerts multifaceted effects on critical cell populations involved in this regenerative process. In BMSCs, EMD enhances the expression of osteogenic markers such as RUNX2, ALP, COL-1, and Osterix, while also promoting cellular adhesion, migration, proliferation, and differentiation. Similarly, PLSCs treated with EMD exhibit increased secretion of TGF-β1, BMP-2, and extracellular matrix components including COL-1, OPN, and OCN, alongside enhanced proliferative and osteogenic capacities. Fibroblasts respond to EMD stimulation by upregulating the production of COL-1, VEGF, and fibronectin, thereby facilitating tissue fibrosis and angiogenesis. In osteoblasts, EMD not only stimulates the expression of TGF-β1, BMP-2, and ALP but also inhibits IL-1β, thereby fostering a pro-regenerative microenvironment that enhances cell adhesion, migration, and mineralization. These coordinated cellular responses collectively contribute to the significant periodontal regenerative potential observed with EMD therapy. ALP, alkaline phosphatase; BMP-2, bone morphogenetic protein 2; BMSCs, bone marrow mesenchymal stem cells; COL-1, collagen type 1; EMD, enamel matrix derivative; IL-1β, interleukin 1 beta; OCN, osteocalcin; OPN, osteopontin; PLSCs, periodontal ligament stem cells; RUNX2, runt-related transcription factor 2; TGF-β1, transforming growth factor beta 1; VEGF, vascular endothelial growth factor.

Periodontal ligament cells (PDLCs) constitute the cellular foundation for EMD-induced periodontal tissue regeneration, predominantly comprising periodontal ligament stem cells (PLSCs), mesenchymal stem cells (MSCs), osteoblasts, fibroblasts, and cementoblasts, each exhibiting distinct biological functions and differentiation potentials (15). In the context of periodontal regeneration, EMD primarily facilitates tissue repair by promoting the directed migration, proliferation, and differentiation of various cell subpopulations within the periodontal ligament (16). *In vitro* investigations indicate that the exposure of PLSCs and primary osteoblasts to EMD, whether in gel or liquid carriers, enhances their proliferative and differentiation capacities. This enhancement is associated with an upregulation of gene expression for transforming TGF-β1 and BMP-2, alongside a downregulation of interleukin-1β (IL-1β) expression (17). EMD-treated periodontal ligament (PDL) cell sheets demonstrate increased thickness and density, characterized by a higher number of cell layers and enhanced extracellular matrix production. These cultures exhibit elevated mRNA expression levels of key osteogenic markers, including COL-1, RUNX2, osteopontin (OPN), OCN, and cementum-associated protein (CAP), alongside improved mineralization capacity during osteogenic differentiation (18). Furthermore, EMD significantly enhances the proliferation and osteogenic differentiation of bone marrow mesenchymal stem cells (BMSCs). Quantitative reverse transcription polymerase chain reaction (qRT-PCR) analyses indicate that EMD supplementation upregulates the expression of essential osteogenic transcription factors, such as RUNX2 and Osterix, as well as other critical markers including alkaline phosphatase (ALP), COL-1, and OCN (19). Spheroid culture experiments further corroborate EMD's capacity to sustain stem cell viability while facilitating osteogenic differentiation, as evidenced by increased ALP activity, mineralization, and RUNX2 mRNA levels (20). Additional investigations confirm that EMD significantly enhance the expression of RUNX2, ALP, and COL-1 at both the gene and protein levels in human BMSCs, thereby promoting their differentiation into osteoblasts and subsequent mineralization (21). Additionally, EMD promotes the proliferation and migration of gingival fibroblasts by increasing COL-1 production in the extracellular matrix and raising the mRNA levels of vascular endothelial growth factor (VEGF) A and fibronectin (22). Endogenous growth factors are crucial for periodontal regeneration. For instance, the application of EMD in conjunction with TGF-β1 significantly augments the proliferation, migration, total protein synthesis, ALP activity, and mineralized nodule formation of periodontal ligament fibroblasts. In contrast, TGF-β1 mainly supports cell adhesion. Collectively, these effects contribute to regeneration of periodontal tissues (23, 24). Figure 1 illustrates the impact of EMD on cells related to periodontal regeneration.

## Clinical applications of enamel matrix derivative in periodontal tissue regeneration

### Enamel matrix derivative and periodontal diseases

The optimal healing outcome for periodontal diseases is characterized by the regeneration of functional periodontal supporting tissues, which include cementum, periodontal ligament, and alveolar bone (5). Clinically, the application of bone grafting materials alone in patients with periodontitis frequently results in suboptimal outcomes, as achieving true periodontal regeneration remains a significant challenge. Early investigations have indicated that the combination of bone grafting materials with EMD markedly enhances clinical outcomes in cases of alveolar bone defects (25). Specifically, when the angle of the alveolar bone defect is ≥40 degrees, the integration of EMD with autogenous bone grafting has been shown to significantly reduce defect depth (26). Notably, a recent systematic review indicated that in the context of periodontal regenerative surgery, the incorporation of bone grafts alongside EMD did not yield additional clinical benefits in periodontal tissue parameters when compared to EMD monotherapy; improvements were observed solely in terms of radiographic defect filling (1). Therefore, EMD is recognized as a crucial component in the regenerative therapy for periodontal defects. Mikami et al. (27) conducted a three-year prospective study involving 253 intrabony defects in 151 patients who received periodontal regenerative treatment (PRT) utilizing EMD. The study systematically evaluated clinical parameters, including probing pocket depth (PPD), clinical attachment level (CAL), and radiographic bone defect depth (RBD). Through multilevel regression analysis adjusted for potential confounders, the researchers observed significant reductions in PPD, as well as increases in both CAL and RBD at the one-year follow-up. Importantly, these therapeutic benefits were either maintained or further enhanced throughout the three-year observation period, with no significant influence of patient age on treatment outcomes. However, for Class II furcation defects, the combined use of biomaterials hydroxyapatite and β-tricalcium phosphate (HA/β-TCP) with EMD did not provide additional clinical advantages (28, 29). Peres et al. conducted a randomized clinical trial to clinically assess the efficacy of HA/β-TCP administered either alone or in conjunction with EMD for the treatment of proximal class II furcation defects. The results indicated that both treatment modalities significantly enhanced clinical parameters, including reductions in probing depth (PD) and increases in attachment and bone levels. However, no statistically significant differences were observed between the treatment groups, and complete furcation closure remained unpredictable (30). One limitation of this study may be the statistical power of the analyses conducted. The estimated standard deviation used for sample size calculation was 1 mm (based on the primary outcome, relative horizontal clinical attachment level, rHCAL); however, the standard deviation observed following the treatments was greater than the estimated value (1.46 mm for HA/β-TCP and 1.58 mm for HA/β-TCP-EMD groups). Consequently, additional randomized controlled trials are warranted to validate the findings of the present study. Furthermore, Limiroli et al. conducted a comparative analysis of the efficacy of a polylactic acid membrane (Guidor) in conjunction with bovine bone graft (Bio-Oss) vs. EMD combined with Bio-Oss for the treatment of mandibular Class II furcation defects. Their findings indicated that both treatment modalities resulted in significant clinical and radiographic improvements over a 24-month period, with EMD yielding marginally superior outcomes (31). Nevertheless, this study is constrained by its limited sample size. In patients with periodontitis exhibiting intrabony defects, the synergistic application of EMD in conjunction with minimally invasive surgical techniques, including modified minimally invasive surgical approaches, modified papilla preservation techniques or, simplified papilla preservation techniques, has been shown to yield substantial clinical enhancements. These enhancements are evidenced by a notable reduction in PD and an increase in CAL (32–34). Furthermore, research indicates that in diabetic patients with well-controlled blood glucose levels, the application of EMD utilizing the simplified papilla preservation flap (SPPF) is particularly effective in reducing PPD and augmenting CAL (35). However, a recent systematic review revealed that EMD combined with minimally invasive periodontal surgery improved gingival recession coverage and bone filling in intrabony defects, though no significant benefits were observed in PD or CAL reduction (36). A notable limitation of this study is the variability in the timing of periodontal therapy phases among participants. For instance, not all studies had patients undergo initial non-surgical therapy prior to the intervention evaluated. The findings would likely be more robust if all patients received EMD treatment during the same phase of periodontal therapy. To further advance the clinical management of periodontal defects, Yang et al. (37) explored the integration of EMD in open flap debridement (OFD). Their findings demonstrated that the combination of EMD and OFD significantly improved clinical attachment levels, reduced PD, and facilitated periodontal regeneration in the treatment of periodontal defects. Furthermore, a case study examining an 11-year follow-up of generalized aggressive periodontitis demonstrated that the application of EMD as a regenerative material for periodontal defects, following open-flap debridement, resulted in significant improvements in the patient's periodontal health. Notably, there was a marked reduction in periodontal pocket depth, a substantial increase in clinical attachment level, and EMD facilitated bone filling in intrabony defects, as well as the regeneration of compromised periodontal tissues (38). Moreover, the utilization of EMD in conjunction with non-surgical periodontal treatment has been shown to enhance treatment outcomes, evidenced by a greater reduction in PPD, a more pronounced increase in CAL, a more effective decrease in bleeding on probing (BOP), and a higher frequency of periodontal pocket closure (39). Recent study has demonstrated that the use of EMD in conjunction with non-invasive flapless surgery for the treatment of intrabony defects significantly enhances both clinical and imaging outcomes. Specifically, this combined approach leads to an increase in CAL, a reduction in PD, and a greater extent of bone defect filling (4). However, recent evidence indicates that the adjunctive use of EMD with non-surgical debridement results in minimal improvement in CAL and does not significantly reduce PPD, modulate inflammation, or offer microbiological benefits compared to debridement alone in residual pockets, indicating limited clinical utility (40). Nonetheless, the restricted sample size may have constrained the detection of additional effects on clinical parameters, cytokine levels, or bacterial profiles. Furthermore, research indicates that the application of EMD in the context of treating gingival recession with the CAF and SCTG markedly improves the root coverage rate. Concurrently, there is a significant increase in the expression of VEGF, which contributes to the overall efficacy of the clinical treatment (41). A systematic review further demonstrated that when EMD was combined with CAF or CAF with connective tissue graft (CTG) for treating maxillary gingival recessions, it significantly reduced recession depth and improved CAL at 6–12 months post-treatment. However, the adjunctive use of EMD did not significantly increase keratinized tissue width (KTW), suggesting that its primary benefits lie in periodontal attachment rather than soft tissue augmentation. For patients seeking optimal root coverage and CAL gain, adjunctive use of EMD with CAF or CAF + CTG may be considered as a viable treatment option (42). Compared to CAF alone, all three treatment modalities—CAF with collagen matrix (CM), CAF with EMD, and CAF with CM + EMD—demonstrated superior clinical outcomes in root coverage. However, regarding complete root coverage (CRC) rates, EMD played a pivotal role, with both the CAF + EMD and CAF + CM + EMD groups achieving the highest performance levels. Moreover, the application of CM slightly but significantly increased gingival thickness which was not observed for CAF+EMD or CAF alone (43). However, when EMD is utilized alongside the semilunar coronally advanced flap (SCPF) for managing gingival recession, it yields superior aesthetic outcomes, characterized by a reduction in scar tissue lines. Nevertheless, in terms of root coverage, its efficacy does not surpass that of the standard SCPF (44). The therapeutic effects of EMD in applications related to periodontal disease are summarized in Table 1.

**Table 1:** The therapeutic effects of EMD in periodontal disease-related applications.

Author and published year	Study type	Defect	Test group	Periodontal parameters	Conclusions	References
De Leonardis et al. 2013	Clinical trial	Intrabony defects	OFD; EMD; EMD+ HA/β -TCP	PD, CAL, RBG, and GR	At 12 and 24 months after treatment, the EMD+ HA/β-TCP group showed significantly greater PD reduction, CAL gain and RBG gain, and less GR increase compared with other groups.	(25)
Matsuura et al. 2024	Cohort study	Intrabony defects	EMD; EMD+ bone grafts	RBD, DA	In EMD group, the 1- and 3-year reduction of RBD showed significant inverse correlations with DA. EMD+ autologous bone grafts might be significantly beneficial for RBD improvement in the case of DA at baseline ≥ 40°.	(26)
Hasuike et al. 2024	System review	Intrabony defects	EMD+ bone grafts	CAL, PD, REC, and Defect fill	The outcome showed no significant differences between EMD and EMD+ bone grafts in terms of CAL, PD and REC. However, EMD+ bone grafts enhanced radiographic filling of bone defects.	(1)
Mikami et al. 2022	Cohort study	Intrabony defects	EMD EMD+ autologous bone grafts	PPD, CAL, RBD	The outcome showed a significant reduction in PPD and gain in CAL and RBD at the 1-year examination, which was sustained or improved 3 years after periodontal regenerative therapy using EMD.	(27)
Queiroz et al. 2016	Clinical trial	Class II furcation defects	EMD; β-TCP/HA; EMD+ β-TCP/HA	RGMP, RVCAL, RHCAL, and PD	No significant intragroup differences were observed for RGMP whereas a significant reduction for PD and a significant gain for RVCAL and RHCAL were observed in all three treatments. However, the outcomes showed no significant difference among the three groups.	(29)
Soares et al. 2020	System review	Class II furcation defects	OFD + β-TCP/HA + EMD	PD, RVCAL, and RHCAL	When comparing OFD + β-TCP/HA with or without EMD, the outcome showed no significantly difference in the treatment of furcation defects in terms of PD, RVCAL and RHCAL.	(28)
Peres et al. 2013	Clinical trial	Class II furcation defects	HA/β -TCP HA/β -TCP+ EMD	PI, GI, PPD, RGMP, RVAL, RHAL, RVBL, and RHBL	Both groups presented improvements after therapies; however, no inter-group differences could be seen in any single parameter. The combination with EMD did not significantly improve the therapeutic effects.	(30)
Limiroli et al. 2023	Clinical trial	Class II furcation defects	Guidor Matrix Barrier+ Bio-Oss EMD+ Bio-Oss	PPD, CAL, REC, KTW and RBG	Both groups showed a significant increase of clinical and radiographic success. EMD+ Bio-Oss showed better clinical outcomes with less complications, although not statistically significant, compared to the other group.	(31)
Windisch et al. 2019, 2022	Clinical trial	Intrabony defects	EMD+ MIST/M-MIST; EMD+ MPP/SPP	PD, CAL, and GR	A significant reduction of PD and a significant gain of CAL were observed in all treatments whereas no statistically significant difference was found in terms of GR.	(32–35)
Estrin et al. 2022	System review	Periodontal defects	MIST MIST + EMD	MIST MIST + EMD	The results showed that EMD + MIST improved REC and BF when compared to MIST without EMD. However, no significant difference in CAL or PD was observed between the two groups.	(36)
Yang et al. 2024	System review	Periodontal defects	OFD + EMD	PD, CAL and GR	OFD + EMD seems to be beneficial in terms of CAL gain, PD reduction, and periodontal regeneration.	(37)
Trikka et al. 2019	Case report	GAgP with Periodontal intrabony defects	OFD + EMD	PD, CAL	The results demonstrated no recurrence of disease within 11-year follow-up. The PD presented satisfactory reduction while the CAL was also improved.	(38)
Chatzopoulos et al. 2022	System review	Periodontal defects	NSPT + EMD	PD, CAL, and BOP	The majority of the included studies demonstrated that NSPT + EMD could lead to significantly treatment outcomes including higher PD reduction, more CAL gain, more robust BOP reduction.	(39)
Aimetti et al. 2024	Clinical trial	Intrabony defects	NSPT(flapless) + EMD; NSPT(flapless)	PD, CAL, and Bone fill	NSPT + EMD showed significantly more PD reduction and CAL increase. In terms of radiographic outcomes, NSPT + EMD yielded a greater defect bone fill than NSPT alone.	(4)
Wehner et al. 2023	Clinical trial	Periodontal defects	Subgingival instrumentation Subgingival instrumentation + EMD	PPD, CAL, BOP, PI, Periodontal pathogen count	Application of EMD as an adjunct to subgingival of residual pockets yielded benefits regarding CAL gain; however, effects on PPD reduction, inflammatory cytokines, and bacterial count were negligible	(40)
Dias et al. 2022	Clinical trial	Gingival recession	CAF + SCTG + EMD; CAF + SCTG	RC, RH, and RW	The use of EMD in root coverage surgeries resulted in a significantly higher RC, as well as significant lesser RH and RW.	(41)
Meza Mauricio et al. 2021	Systematic review	Gingival recession	CAF CAF + EMD CAF + CTG CAF + CTG + EMD	GR, KTW, CAL	The adjunctive application of EMD in the treatment of GR in maxillary teeth either with CAF or CTG provided moderate certainty evidence in favor of their use for reduction in GR and gain in CAL at 6 and 12 months.	(42)
Sangiorgio et al. 2017	Clinical trial	Gingival recession	CAF CAF + CM CAF + EMD CAF + CM + EMD	GR, PD, CAL, KTW, and KTT	Compared with CAF alone, the other 3 approaches are superior for root coverage. Nevertheless, CAF + EMD and CAF + CM + EMD obtained highest levels of complete root coverage.	(43)
Franca-Grohmann et al. 2019	Clinical trial	Gingival recession	SCPF SCPF + EMD	RH, RW, WKT, TKT, PD, CAL	The addition of EMD provides significantly better esthetics to SCPF. However, SCPF + EMD is effective but not superior to SCPF for root coverage after 12 months. No significant differences were showed between guoups for periodontal parameters.	(44)

PD, probing depth; CAL, clinical attachment level; RBG, radiographic bone gain; RBD, radiographic bony defect depth; GR, gingival recession; DA, bone defect angle; REC, recession change; RGMP, relative gingival margin position; RVCAL, relative vertical attachment level; RHCAL, relative horizontal attachment level; PI, plaque index; GI, gingival index; RVBL, vertical bone level; RHBL, horizontal bone level; KTW, keratinized tissue width; KTT, keratinized tissue thickness; BOP, bleeding on probing; RC, recession coverage; RH, recession height; RW, recession width; HA/β-TCP, hydroxyapatite and β-tricalcium phosphate; CAF, coronally advanced flap; CTG, connective tissue graft; SCTG, subepithelial connective tissue graft; OFD, open flap debridement; MIST, minimally invasive surgical technique; Bio-Oss, heterologous bone; M-MIST, modified minimally invasive surgical technique; MPPT, modified papilla preservation technique; SPPT, simplified papilla preservation technique; NSPT, non-surgical periodontal treatment; GAgP, generalized aggressive periodontitis.

### Enamel matrix derivative and dental implantation

EMD has been demonstrated to facilitate periodontal tissue regeneration, repair damaged bone tissue, and inhibit further bone resorption. Its application has been extensively researched and implemented in the domain of dental implantation. The establishment of osseointegration at the implant-bone interface is critical for the success of implant restoration. This process entails direct structural contact between the surface of the loaded implant and the bone tissue, without any intervening tissue, thereby facilitating for the continuous transmission and dispersion of the implant's load within the bone tissue. *In vitro* studies have indicated that EMD stimulation enhances osteoblast activity on the implant surface, as evidenced by increased osteocalcin production, elevated ALP activity, and upregulated mRNA expression of osteoprotegerin (OPG), all of which positively influence osseointegration at the implant-bone interface (45). Additionally, EMD has been shown to promote the proliferation, adhesion, and migration of osteoblasts on titanium surfaces in a concentration-dependent manner (46). There is a growing body of research on EMD in the context of dental implantation. EMD has been shown to significantly enhance the proliferation and osteogenic differentiation of periodontal ligament stem cells (PDLSCs) on the surface of titanium implants by activating the Akt/mTOR signaling pathway, thereby providing a foundational experimental basis for its application in peri-implant bone regeneration (47). Peri-implantitis is a plaque-associated pathological condition occurring in tissues around dental implants, characterized by inflammation in the peri-implant mucosa and subsequent progressive loss of supporting bone (48, 49). EMD exhibits anti-inflammatory and immunomodulatory properties, effectively inhibiting the activity of inflammatory cells and the release of inflammatory mediators, which subsequently reduces the inflammatory response surrounding the implants (50). Recent studies indicate that the combined treatment of peri-implantitis with EMD during surgical intervention yields a 100% implant survival rate at three years and an 85% survival rate at five years. The adjunctive use of EMD during surgery is positively correlated with implant survival; however, further validation through larger-scale studies is warranted (51). A case-series study examining the application of EMD in the surgical management of peri-implantitis demonstrated that the utilization of EMD during surgical procedures is associated with a notably high survival rate of implants affected by peri-implantitis. Furthermore, there was a statistically significant improvement in postoperative PD, accompanied by a reduction in BOP (52). A randomized clinical trial found that adding EMD to surgery for peri-implantitis significantly improved outcomes, with better marginal bone levels and a shift towards Gram-positive/aerobic bacteria at 12 months, indicating EMD may enhance bone regeneration and microbial profiles (53). Furthermore, research has demonstrated that the combined use of deproteinized bovine bone mineral (DBBM) and EMD in alveolar ridge preservation following tooth extraction significantly enhances new bone formation during socket healing, thereby creating more favorable conditions for subsequent implant placement (54). Additionally, Wen et al. (55) performed a partial transverse implantation of 30 Straumann BL implants in the posterior mandibles of 15 rabbits. Following a 10-week healing period, histological analysis of the retrieved specimens was conducted to assess new bone formation. The results further corroborated that the combined application of EMD facilitated an increase in both vertical bone height and bone density. Ikawa et al. (56) investigated the use ofEMD as an adjunctive material in natural bovine bone grafting for peri-implant bone defects. Their findings demonstrated that EMD significantly enhanced new bone formation and osseointegration in these defects. Specifically, the new bone area, bone-to-implant contact (BIC), and first bone-to-implant contact (fBIC) were all markedly greater than those observed in the control group. A recent narrative review on the application of EMD in dental implantation further supports its promising potential for use in implant placement and bone regeneration in peri-implant bone defects. Nevertheless, additional randomized clinical trials are required to thoroughly assess its efficacy (57). Furthermore, EMD not only influences bone tissue but also modulates the behavior of soft-tissue cells (58). It has been shown to promote the proliferation and migration of fibroblasts, enhance collagen synthesis, and facilitate the formation of a healthy soft-tissue seal around the implant, thereby reducing the risk of bacterial invasion in the surrounding tissues (22). Furthermore, an experimental study investigating EMD's effects on oral mucosal wound healing in rats demonstrated that EMD-treated surgical sites exhibited significantly enhanced tissue regeneration, as evidenced by: (1) increased proliferating cell numbers, (2) greater vascular density, and (3) elevated collagen deposition. Molecular analyses revealed upregulated mRNA expression of key healing mediators—including IL-1β, MMP1, TGFβ1, TGFβ2, VEGF, versican, and fibronectin—suggesting EMD accelerates oral mucosal wound repair through multifaceted modulation of the healing cascade (59). A split-mouth randomized controlled trial with 30 patients and 60 implants found that using EMD during single-stage implant placement in healed alveolar ridges significantly improved early peri-implant soft tissue healing. EMD-treated sites showed better healing index scores, reduced probing depth and bleeding, and increased keratinized tissue width compared to controls. Patients also reported less pain, reduced swelling, and higher aesthetic satisfaction with EMD. These results confirm that EMD can effectively enhance early soft tissue healing after implant placement (60). However, a recent randomized clinical trial on the efficacy of EMD in the reconstructive surgical therapy of peri-implantitis failed to demonstrate the beneficial effects of adjunctive use of EMD. The reasons may be related to the imbalance in baseline PPD and MBL levels between the two groups, the uneven distribution of drop-outs, the sample size calculation based on radiographic MBL changes (inconsistent with the conventional design of randomized controlled trials), and the generic use of systemic antibiotics (61). Despite the promising potential of EMD in the domain of dental implantation, further clinical investigations are necessary to comprehensively assess its long-term effects and safety, as well as to optimize its application methods and strategies.

### Enamel matrix derivative and tooth replantation

Tooth replantation is a therapeutic procedure whereby a dislodged tooth, displaced for various reasons, is reinserted into its original alveolar socket. The success of this intervention is contingent upon several critical factors, including the prevention of replacement root resorption, the promotion of periodontal tissue healing, and the reattachment of the root to the alveolar bone. Given that EMD has been shown to facilitate periodontal tissue regeneration, it has been incorporated into research concerning tooth replantation and transplantation. During the replantation process, root resorption emerges as a significant determinant of the long-term prognosis for replanted teeth. EMD has the capacity to modulate cellular behavior, inhibit osteoclastic activity, and mitigate root resorption, thereby enhancing the prospects for the long-term retention of replanted teeth (50). Al-Hezami et al. (62) conducted a case study involving a 15-year-old female patient diagnosed with suppurative apical periodontitis of the maxillary lateral incisor, attributed to a radicular groove deformity. The treatment regimen comprised a combination of root canal therapy, intentional replantation, and the application of Emdogain. Over a follow-up period of four years, the patient reported a significant improvement in comfort, accompanied by a marked regression of periapical pathology. Furthermore, a two-year prospective case series study investigating the efficacy of Emdogain in conjunction with intentional replantation for the management of hopeless teeth with endodontic-periodontal lesions revealed that, after two years, 16 cases exhibited successful clinical healing. This was evidenced by a reduction in PD, an increase in CAL, and radiographic assessments indicating no root resorption and an enhancement in bone levels. The differences observed compared to baseline values were statistically significant (63). Mohamed et al. (64) conducted a systematic review to investigate the efficacy of EMD in the repair of replanted human teeth. Within the review, two controlled trials demonstrated that EMD treatment significantly reduced root resorption in replanted teeth and enhanced the healing of the periodontal ligament when compared to the control group (65, 66). Nevertheless, the limited number of studies included in the review renders the precise efficacy of EMD inconclusive. Notably, a recent meta-analysis indicated that, in comparison to the absence of EMD, its application did not confer significant advantages in restoring normal periodontal ligament healing in replanted teeth. However, as a bioregulatory factor with diverse functions, EMD may play a role in mitigating the progression of root resorption and improving overall prognosis. Based on current evidence, we hypothesize that: (1) a critical number of viable periodontal ligament cells (PDLCs) is essential for successful tissue regeneration; (2) EMD has limited ability to restore function in severely damaged PDLCs, limiting its effectiveness in tooth replantation; and (3) when sufficient functional PDLCs are present, EMD significantly improves ligament reattachment and root coverage, optimizing periodontal repair (50). The clinical implications of EMD in dental implantation and tooth replantation are detailed in Table 2.

**Table 2:** The clinical effects of EMD in dental implantation and tooth replantation.

Author and published year	Study type	Defect	Test group	Periodontal parameters	Conclusions	References
Isehed et al. 2018	Clinical trial	Peri-implantitis	Surgical treatment + EMD; Surgical treatment	Implant loss, BL change	In the EMD group, 100% implants survived at the 3-year follow-up and 85% implants survived at the 5-year follow-up which were more than the control group. However, the greater gain of BL in EMD group was not statistically significant from the control group.	(51)
Wilson et al. 2023	Case series	Peri-implantitis	Surgical intervention + EMD	MPD, DPD, BOP	The results of this case series demonstrate a high level of survival (94%) of implants when applied with EMD and a highly significant improvement in PD and reduction in BOP when EMD was used.	(52)
Isehed et al. 2016	Clinical trial	Peri-implantitis	OFD+ EMD; OFD	BL change	Adjunctive EMD to surgical treatment of peri-implantitis was associated with increased marginal BL 12 months after treatment. In multivariate modelling, increased marginal BL at implant site was significantly associated with EMD.	(53)
Mercado et al. 2021	Clinical trial	Maxillary anterior ridge preservation after extraction	DBBMC + EMD; DBBMC	%NB, %RG	The DBBMC + EMD group showed significantly increased new bone formation(%NB) and less residual graft(%RG) compared to the DBBMC control group.	(54)
Wen et al. 2016	Animal study	Peri-implant bone regeneration	BCPT1/BCPT2/DBBM + EMD; BCPT1/BCPT2/DBBM	Bone height, fBIC, BA/TA	The bone height was higher for the treatments with EMD than without EMD, but differences were not statistically significant. The release of EMD to a bone-level implant consistently regenerated the greater fBIC and bone density (BA/TA) along the length of the implant.	(55)
Ikawa et al. 2019	Animal study	Peri-implant bone defects	NBB NBB + EMD	BIC, fBIC	New bone area, BIC and fBIC in the NBB and NBB + EMD groups were significantly greater than in the control group. Further, adjunct use of EMD appears to further enhance bone formation and osseointegration.	(56)
Alberti et al. 2021	System review	Peri-implant bone defects	EMD/EMD+ Biomaterials	Bone formation, BIC	A sparse evidence was found on the efficacy of the use of EMD for increasing bone formation and as an adjunct for the treatment of peri-implant defects. In general terms, EMD could improve bone to implant contact (BIC) in immediately positioned implants.	(57)
Cardaropoli et al. 2024	Clinical trial	Wound of peri-implant soft tissues	EMD	Soft tissue healing index (HI)	The use of EMD provided better outcomes. It's beneficial to improve and accelerate soft tissue wound healing around implants.	(59)
Regidor et al. 2025	Clinical trial	Peri-implantitis	Access flap +bone graft + resorbable membrane +EMD	PPD, BOP, SOP, KM and MBL	The addition of EMD didn't result in any statistically significant improvement in clinical or radiographic outcomes between the two groups.	(61)
Al-Hezaimi et al. 2009	Case report	Pulp necrosis with suppurative apical periodontitis	Endodontic therapy + IR + EMD	Periradicular radiolucency, PPD	Four-year follow-up radiograph showed substantial decrease in size of the periradicular radiolucency. Moreover, PPD also showed substantial reduction. The tooth is asymptomatic, and the patient is comfortable.	(62)
Saida et al. 2018	Case series	Hopeless teeth associated with endodontic-periodontal lesions	IR+ EMD	PD, CAL, radiographic bone level	Intentional replantation (IR) + EMD provided significant reduction in PD, gain in CAL, and gain in radiographic bone level compared to baseline values.	(63)
Mohamed et al. 2019	System review	Tooth replantation	EMD	Root resorption, periodontal healing	Among which two controlled trials found significantly reduced resorption of replanted teeth and improved the healing of periodontal ligament. However, the number of publications were limited to provide effective evidence for EMD in supporting healing of replanted teeth.	(64)
Lin et al. 2024	Review	Tooth replantation	EMD	Periodontal healing, extraction risk	EMD may not result in a numerical increase in normal periodontal healing for replanted teeth. However, it may arrest the progression of resorption, thus reducing the extraction risk in the early stage.	(50)

PD, probing depth; MPD, mean probing depth; DPD, deepest probing depth; CAL, clinical attachment level; BOP, bleeding on probing; SOP, suppuration on probing; KM, the width of keratinized mucosa; MBL, marginal bone levels; BL, bone level; BIC, bone to implant contact; fBIC, first bone to implant contact; BA/TA, bone density; DBBMC, deproteinized bovine bone mineral with 10% collagen; BCPT1, Macro-structuring BiPhasicCaPST; BCPT2, Micro-structuring BiPhasicCaPST; NBB, natural bovine bone; IR, intentional replantation; OFD, open flap debridement; NB, new bone formation; RG, less residual graft.

### Mechanisms of enamel matrix derivative in promoting periodontal tissue regeneration

**Figure 2:**
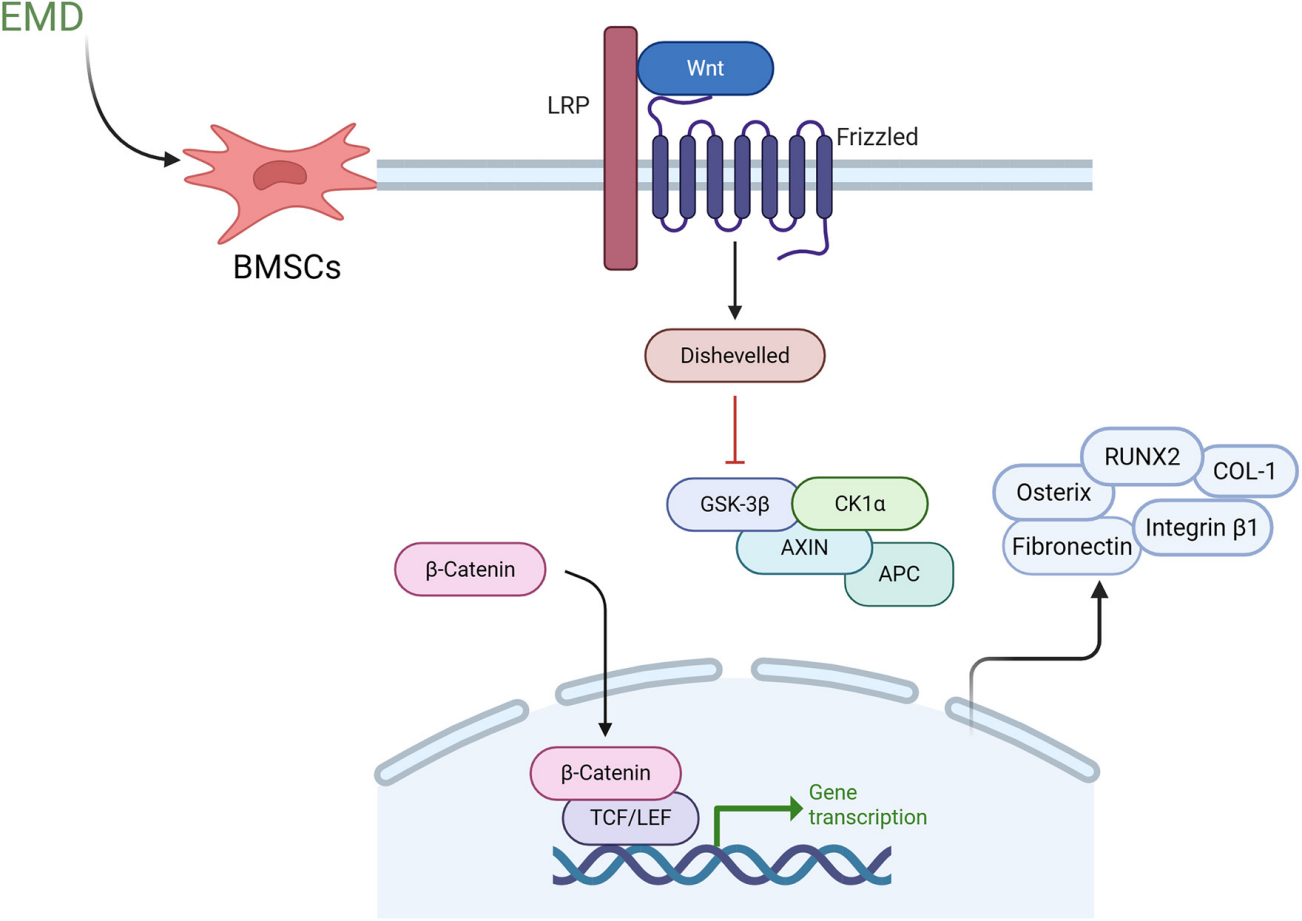
Proposed mechanism of enamel matrix derivative in periodontal regeneration through Wnt/β-catenin signaling pathway activation. In the presence of EMD, BMSCs exhibit enhanced activation of Wnt signaling, which occurs via the binding of Wnt ligands to upregulated membrane receptor complexes that include lipoprotein LRP and Frizzled receptors. This interaction triggers the activation of the intracellular protein Dvl, leading to the inhibition of the β-catenin degradation complex comprised of GSK3-β, Axin, APC and CK1α. As a result, β-catenin is stabilized in the cytoplasm. The accumulated β-catenin subsequently translocates to the nucleus, where it interacts with TCF/LEF transcription factors to upregulate downstream target genes, including osteogenic markers such as Osterix, RUNX2, and COL-1, as well as adhesion molecules like integrin beta and fibronectin. This cascade ultimately promotes cellular proliferation, differentiation, and maturation processes. EMD, enamel matrix derivative; BMSCs, bone marrow mesenchymal stem cells; LPR, lipoprotein receptor-related protein; Dvl, dishevelled; GSK-3β, glycogen synthase kinase 3 beta; APC, adenomatous polyposis coli; CK1α, casein kinase 1 alpha; RUNX2, runt-related transcription factor 2; COL-1, collagen type 1; TCF/LEF, T-cell factor/lymphoid enhancer factor.

Cell differentiation is a multifaceted and dynamic process that involves various growth factors and signaling pathways. While the mechanisms by which enamel matrix proteins facilitate periodontal regeneration are not yet fully elucidated, recent studies have explored potential pathways, which will be discussed in detail below. Early investigations have identified the classical Wnt signaling pathway as a significant contributor to periodontal regeneration. This pathway has been shown to promote the differentiation of periodontal ligament fibroblasts into the osteoblast lineage while simultaneously stimulating the expression of osteogenic transcription factors (67). Furthermore, the classical Wnt/β-catenin signaling pathway is recognized as a critical pathway for the osteogenic differentiation of BMSCs (68). EMD has been found to enhance the proliferation and differentiation of BMSCs, with its mechanism potentially linked to the activation of the Wnt/β-catenin signaling pathway (19). As illustrated in Figure 2, Wnt signaling is initiated when Wnt ligands bind to a receptor complex at the cell surface, comprising lipoprotein receptor-related protein (LRP) and Frizzled receptors. This interaction activates the cytoplasmic protein Dishevelled (Dvl), which subsequently inhibits the β-catenin degradation complex, consisting of glycogen synthase kinase 3 beta (GSK3β), Axin, adenomatous polyposis coli (APC), and casein kinase 1 alpha (CK1α). As a result, β-catenin accumulates in the cytoplasm and translocates to the nucleus, where it interacts with T-cell factor/lymphoid enhancer factor (TCF/LEF) transcription factors to regulate the expression of downstream target genes. This signaling cascade ultimately promotes cellular proliferation, differentiation, and maturation processes (69, 70). In the presence of EMD, reverse transcription quantitative polymerase chain reaction (RT-qPCR) analyses indicate that the expression levels of osteogenesis-related transcription factors, including Osterix, RUNX2, and COL-1, are significantly upregulated. Additionally, the expression of adhesion-related transcription factor genes, such as Integrin β1 and Fibronectin, is also elevated. Western blotting and RT-qPCR analyses further demonstrate an increase in both protein and mRNA levels of β-catenin (71). Liu et al. (72) employed microRNA microarray technology in conjunction with real-time quantitative PCR (qPCR) to demonstrate that the expression of miR-30a significantly increases during the cementogenic differentiation of PLSCs in response to EMD. This upregulation of miR-30a notably enhances the expression of cathepsin K (CTSK). Furthermore, the inhibitory modulation of the Wnt/β-catenin signaling pathway markedly attenuates the regulatory influence of miR-30a on CTSK expression. The results of this study indicate that EMD facilitates the cementogenic differentiation of PLSCs by elevating miR-30a levels, which in turn enhances the expression of the regulatory factor phosphorylated GSK-3β and the core regulatory factor activated β-catenin. Additionally, the activation of the Wnt/β-catenin signaling pathway is implicated in this process. Other research has indicated that amelogenin can specifically bind to glucose-regulated protein 78 (Grp78), a receptor located on the cell membrane, thereby promoting the internalization of amelogenin into the cell. This interaction significantly enhances cell migration without impacting cell proliferation (73). The mitogen-activated protein kinase (MAPK) pathway constitutes a critical mechanism for cell proliferation and osteogenic differentiation. This pathway encompasses c-Jun N-terminal kinase (JNK), extracellular signal-regulated kinase (ERK), and p38 kinase (p38), which facilitate the transduction of extracellular signals into cells and the nucleus, thereby eliciting a range of biological effects (14). Early investigations into the mitogenic response of PDLCs to EMD revealed that EMD activates the ERK1/2 signaling pathway via the EMD-specific receptor tyrosine kinase (RTK), thereby initiating cell mitotic signals (74). Furthermore, recent research has demonstrated that EMD promotes the mitosis of periodontal ligament fibroblasts (PDLFs) through the ERK1/2 pathway (75). Additionally, a study examining the effects of synthetic oligopeptides (SP) derived from EMD on the proliferation and osteoblast differentiation of human MSCs indicated that the extracellular signal-regulated kinase (ERK) is involved in the cell proliferation and osteoblast differentiation induced by SP. SP has been shown to enhance the proliferation, differentiation into osteoblasts, and mineralization of MSCs. Conversely, the application of ERK1/2 inhibitors attenuates these effects, indicating that SP may facilitate cell proliferation and osteoblast differentiation in human MSCs via the ERK signaling pathway (76). Additionally, the p38 MAPK pathway has been implicated in the upregulation of matrix metalloproteinase-2 (MMP-2) in osteoblasts activated by EMD. MMP-2 subsequently contributes to the regeneration of periodontal tissue by degrading matrix proteins within the periodontal connective tissue (77). Furthermore, interactions between the MAPK and Wnt/β-catenin signaling pathways have been established, with evidence suggesting that the classical Wnt/β-catenin pathway is modulated by the MAPK pathway, which plays a pivotal role in intracellular signal transduction (78). Recent investigations have also demonstrated that extracellular matrix proteins (EMP) can inhibit the expression of inflammatory mediators in bone marrow stromal cells stimulated by IL-1β and tumor necrosis factor-alpha (TNF-α). This anti-inflammatory effect may be mediated through the activation of the TGF-β-related signaling pathway (11). Despite significant efforts to elucidate its mechanisms, the specific signaling pathways through which EMD facilitates periodontal tissue regeneration remain inadequately understood, necessitating further in-depth investigation.

## Discussion and future perspectives

Periodontal tissue defects resulting from periodontal diseases and their treatment continue to be a central area of research within the field. Over the past few decades, numerous innovative strategies and products have been developed for the repair and regeneration of periodontal defects, with EMD emerging as one of the most extensively utilized biological agents. EMD, a crucial molecule in tooth development, promotes local growth factor expression, extracellular matrix deposition, mineralization, and wound healing, thereby exhibiting considerable potential in oral medicine (20, 79, 80). Research indicates that the adjunctive application of EMD following non-surgical scaling and root planing (SRP) reduces fibrinolytic activity, diminishes inflammatory cytokine levels, significantly decreases PD, and enhances CAL, thereby promoting improved healing of periodontal pockets (81). However, some studies have reported less favorable outcomes with EMD application. In patients with moderate-to-severe periodontitis, non-surgical SRP augmented with EMD did not yield additional benefits in PD or CAL improvement; however, overall periodontal health was enhanced, as evidenced by a reduction in BOP and an increased prevalence of healthy periodontal pockets (82). Consequently, it is imperative to conduct longitudinal histological studies to assess the efficacy of EMD in conjunction with non-surgical interventions for periodontal tissue regeneration. Moreover, future investigations should incorporate blinded control groups and utilize calibrated examiners to enhance the reliability and validity of the findings. Furthermore, a recent trial reported only marginal gains in CAL with EMD during non-surgical SRP for residual pockets, with no significant effects observed on PD, inflammatory markers, or bacterial load (40). These inconsistencies may be attributed to incomplete removal of blood from root surfaces, which can affect EMD adsorption, or variations in the efficacy of calculus removal (36). Other studies employing deep sequencing approaches have investigated alterations in the periodontal microbiome following EMD treatment. The results demonstrate that EMD therapy can significantly modify the dysbiotic subgingival microbiota, characterized by a reduction in pathogenic bacterial abundance and concomitant increase in commensal microorganisms. However, further research is warranted to elucidate the mechanistic relationship between these microbial shifts and periodontal regeneration outcomes (83). In cases of deep periodontal pockets with intrabony defects, modified minimally invasive surgery alone has demonstrated comparable short- and long-term outcomes to regenerative combination therapies, while also incurring lower costs; however, larger independent studies are necessary to validate these findings (84). A systematic review indicated that the application of EMD in conjunction with bone substitutes resulted in significantly greater CAL gains in intrabony defects with follow-up periods of one year or more; however, it did not demonstrate any additional advantages for furcation defects in terms of CAL or PD reduction (29, 85). When comparing sites treated with EMD to those treated only with bone substitutes or EMD plus bone substitutes, differences in histological healing patterns should be noted (29). The reasons for the lack of effect at certain sites are unclear, but it is speculated that the microbiome and molecular signature of furcation defects differ significantly from interproximal sites. This suggests that the unique anatomy of furcations may influence microbial diversity and host response (85). *In vitro* investigations have shown that EMD possesses the capacity to promote robust directional migration in keratinocytes and osteoblasts, enhance cellular viability, and exert anti-inflammatory effects (12). In a randomized clinical trial with 44 patients, EMD treatment for palatal mucosal excision wounds showed no significant differences from the control group in wound area, healing time, pain, or analgesic use during the 90-day follow-up, with both groups achieving complete wound closure by 30 days. Although EMD affected certain inflammatory markers, including monocyte chemoattractant protein-1 (MCP-1), macrophage inflammatory protein-1*α* (MIP-1α), matrix metalloproteinase-9 (MMP-9), and tissue inhibitor of metalloproteinases-2 (TIMP-2), these changes did not lead to clinical benefits, concluding that EMD offers no advantage in palatal wound healing (86). However, this study is subject to several limitations. Notably, there is currently insufficient data regarding the optimal dosage and application frequency of EMD for optimal soft tissue healing. Exploring various concentrations or multiple applications may uncover additional benefits of EMD in excisional wound repair. Additionally, the absence of a placebo gel in the control group represents another potential limitation. A single-blind randomized controlled study found that using EMD with the MCAT technique and SCTG for gingival recessions did not significantly impact early wound healing or clinical outcomes (87). The results are consistent with those of a 3-year longitudinal retrospective cohort study based on the population (88). Recent findings indicate that the MCAT technique with SCTG is highly effective for treating RT1 and RT2 recession defects, but adding EMD does not significantly improve root coverage or periodontal health. This may be due to limited root access during tunnel preparation and possible blood contamination affecting EMD application. However, EMD-treated sites do experience less postoperative pain in the early healing stages (89). Consequently, further mechanistic studies are warranted.

Initial research examining the effects of EMD concentration indicated that high concentrations (75–100 μg/ml) inhibited the activity of PDLFs over time, whereas lower concentrations (25–50 μg/ml) stimulated their activity (90). Similarly, EMD at concentrations of 25–50 μg/ml significantly enhanced the proliferation of BMSCs, with 25 μg/ml being identified as the most effective concentration (19). Recently, under high-glucose conditions (25 mmol/L), a concentration of 75 μg/ml EMD was found to optimally induce BMSCs proliferation and osteogenic differentiation (71). In the context of PDLSCs cultured on titanium surfaces, a concentration of 30–60 μg/ml of EMD was found to significantly enhance ALP activity, mineralization, and the expression of RUNX-2 and OCN (47). The optimal concentration of synthetic peptides (SP) derived from EMD is contingent upon the specific cell type, with 10 ng/ml being effective for MSCs and 100 ng/ml for PDL fibroblasts and stem cells (76). These observations highlight the importance of context and cell type in determining the appropriate dosing of EMD. However, there is insufficient clinical research on the best EMD dosage and application frequency for periodontal intra-bony defects or furcation involvement. Current trials mainly compare outcomes with or without EMD. Future studies should include well-designed randomized controlled trials to assess the impact of varying EMD concentrations on specific periodontal issues. Furthermore, the efficacy of EMD is influenced by the carrier systems utilized; for instance, the liquid formulation of EMD (Osteogain®) demonstrates comparable effectiveness to gel-based EMD in stimulating osteoblasts and PDL cells (17). *In vitro* studies indicate that barrier membranes combined with Osteogain® promote osteoblast adhesion, differentiation, and mineralization (91). However, additional animal studies are warranted to optimize delivery methods and concentrations for effective tissue regeneration.

By elucidating the composition, biological properties, and mechanisms of EMD, as well as refining clinical protocols, EMD-based therapies have the potential to provide more effective solutions for periodontal and implant-related challenges, thereby advancing the field of oral medicine. Nevertheless, significant issues remain unresolved, underscoring the need for intensified basic and clinical research to fully harness the potential of EMD. Despite notable advancements in the applications of extracellular EMD, several critical challenges remain. These challenges include an incomplete understanding of the molecular mechanisms underlying EMD, particularly in the contexts of cell signaling and gene regulation, as well as the absence of standardized clinical protocols governing dosing, delivery, and treatment timing. Future research endeavors should capitalize on advanced technologies, such as single-cell RNA sequencing and CRISPR-Cas9, to further elucidate the mode of action of EMD. Additionally, large-scale clinical trials are imperative to optimize therapeutic parameters. The innovation of next-generation EMD formulations—including nanoparticle carriers, 3D-printed scaffolds, and smart hydrogels—has the potential to significantly enhance bioavailability and targeting efficacy. Moreover, expanding the applications of EMD to areas such as maxillofacial reconstruction, management of oral mucositis, and peri-implant tissue engineering may unveil new therapeutic avenues. Addressing these priorities through a synergistic approach that integrates basic and clinical research will be crucial for fully realizing the potential of EMD in the field of regenerative dentistry.

Notably, this review has limitations. Although clinical evidence supports EMD's therapeutic potential, several studies were industry-funded, which may affect the interpretation of the findings despite their adherence to methodological standards.
